# Production of Pitch from Coal Tar of the Coke Chemical Production “Qarmet”

**DOI:** 10.3390/molecules30071441

**Published:** 2025-03-24

**Authors:** Aigul T. Ordabaeva, Zainulla M. Muldakhmetov, Mazhit G. Meiramov, Sergey V. Kim, Zhenisgul I. Sagintaeva

**Affiliations:** 1 Institute of Organic Synthesis and Chemistry of Coal of Kazakhstan Republic, Alikhanov Str., 1, Karaganda 100012, Kazakhstan; 2 Laboratory of Thermochemical Processes, Zh. Abishev Chemical-Metallurgical Institute, Karaganda 100009, Kazakhstan

**Keywords:** pitch, coal tar, distillation, chromatography

## Abstract

Medium-temperature pitch was obtained by vacuum distillation of coal tar from the Qarmet coke chemical production. To determine the composition of the organic constituent of the Qarmet coal tar, the component composition was analyzed via gas–liquid chromatography (GLC) methods. The analysis of the component composition of the organic component of coal tar showed that the content of naphthalene and its derivatives is 37.57%, acenaphthene—2.10%, dibenzofurane—3.60%, fluorene—4.63%, phenanthrene—8.63%, anthracene—2.29%, fluoranthene—4.56%, and pyrene—2.84%. It was found that in the obtained pitch sample, indicators such as the content of insoluble in toluene (41.86%), the softening temperature (85–91 °C), and the yield of volatile substances (1.5%) are more consistent with the standards for electrode pitch grade “V”. The composition of fractions in baking soda, soluble in toluene and quinoline, has been established. In the fractions of pitch soluble in toluene, 11 components were identified, the main of which re fluoranthene (11.71%), pyrene (10.13%), phenanthrene (7.31%), and benzopyrene (4.84%). Thus, based on the analyses carried out, it was found that the Qarmet resin is suitable for obtaining a V-grade electrode pitch, which can be used in the aluminum industry.

## 1. Introduction

Coal tar is a complex multicomponent product of coal processing, which is used in various industries. Due to its unique composition, coal tar serves as a source of a wide range of chemical compounds, including aromatic, heterocyclic, and polycyclic hydrocarbons [1], as well as oxygen-containing compounds such as ketones, phenols, acids, and pitches [2]. Efficient processing of coal tar makes it possible to minimize the environmental risks associated with its storage and disposal by obtaining high-value products with added value [3].

Thus, it was found in [4] that the hydrogenation of coal tar in the presence of the MoNi/γ-Al_2_O_3_ catalyst at a temperature of 360 °C and a pressure of 6–10 MPa and the further hydrogenation of the obtained products in the presence of the WNiP/γ-Al_2_O_3_-USY catalyst at a temperature of 380 °C and a pressure of 6–10 MPa makes it possible to obtain fuel, consisting of a gasoline fraction (30.8%) and a diesel fraction (60.8%). It was noted that the resulting fuel has a low content of sulfur (<50 ppm) and nitrogen (<10 ppm).

The study of the physico-chemical properties of coal tar plays a key role in the development of methods for its processing to extract valuable components [5] and in determining the areas of its potential application. Thus, a study [6] showed that coal tar is a promising cooling agent for coal gasification processes. However, due to its tendency to increase the viscosity at a temperature of 250 °C caused by thermal polymerization of the olefins and phenolic compounds contained in it, modification with the introduction of polymerization inhibitors is necessary to increase thermal stability.

The coal pitch remaining after the distillation of coal tar is also a valuable raw material that is used to produce electrode materials, binders, carbon fibers, structural carbon materials, etc. [7].

Efficient processing of coal pitch requires detailed consideration of its composition, including the content and distribution of polycyclic aromatic hydrocarbons by molecular weight [8]. The fractional composition of pitch has a significant effect on its physico-chemical properties, such as heat resistance, porosity, and specific surface area, which determines its suitability for various industrial applications [9]. The composition of coal pitch is mostly represented by a set of multinucleated aromatic and heterocyclic hydrocarbons, including compounds formed as a result of polycondensation and polymerization of these hydrocarbons [10,11,12,13,14].

The qualitative parameters of the coal pitch remaining after the coal tar is dispersed also directly depend on the coal metamorphism and the coking regime [15]. For example, in the work of Garcia et al., it was found that low-temperature coal pitch (CTP1) forms a mesophase at a lower temperature and at a higher rate compared to high-temperature coal (CTP2) and petroleum (PP) pitches due to the high content of oxygen-containing functional groups and aliphatic substituents, contributing to an increase in the coagulation rate of mesophase spheres [16]. The qualitative characteristics of coal pitch, including the softening temperature, volatile matter content and carbon yield, as well as the ability to form a mesophase, directly depend on the conditions of its processing, including temperature, holding time, and the atmosphere of the heat treatment process, which significantly depend on the conditions of heat treatment, such as temperature, holding time, and gas environment. As shown in [17], heat treatment of coal pitch at 430 °C for 4–5 h in a nitrogen atmosphere leads to a significant increase in carbon yield (up to 69%) and an increase in mesophase content up to 65%, the removal of volatile components, as well as the formation of large polycyclic structures due to dehydropolymerization of aromatic compounds, an increase in the carbon/hydrogen (C/H) ratio, and an increase in structural order, which makes this material promising for further use in the production of carbon materials, including electrodes and composite materials.

Thus, the properties of coal pitch and its suitability for further processing are determined by the composition of the feedstock and the modes of thermal treatment [18].

The purpose of this study is to determine the component composition of the coal tar accumulated in the chemical waste of the Qarmet coke plant and to evaluate its suitability for processing to produce valuable products such as coal pitch. The work is aimed at studying the physico-chemical properties of the resin and the pitch obtained from it to assess their suitability for use in the production of electrode coke.

The analysis of the resin composition from the waste of the Qarmet coke production was carried out using analytical methods of gas–liquid chromatography (GLC). The data obtained make it possible to more accurately assess the potential value of resin as a raw material source and suggest effective ways to process it.

## 2. Results and Discussion

To determine the organic component of the Qarmet coal tar, samples were taken from two landfills of the Qarmet coke chemical production and a resin sample directly from the coke production. The analysis of the component composition of Qarmet coal tar from landfills № 1, № 2, and coal tar obtained directly during the production of coke was obtained using gas–liquid chromatography (GLC).

Various methods for analyzing the composition of coal tar are described in the literature, such as IR spectroscopy (FTIR), nuclear magnetic resonance spectroscopy (HNMR), high-performance liquid chromatography (HPLC), and mass spectrometry (MS). HNMR makes it possible to determine the ratio of aromatic and aliphatic structures, FTIR is used to identify the presence of functional groups and certain structural features (for example, aromatic systems with a certain arrangement of hydrogen atoms), gas chromatography (GC) allows for determining the composition of light aromatic hydrocarbons and the quantitative ratios of various components, and gas chromatography–mass spectrometry (GC-MS) allows for the identification of individual organic compounds [2,12,13,15].

In our study, the gas–liquid chromatography (GLC) method was chosen for the analysis of resin composition for the following reasons: this method is widely used for the analysis of organic mixtures and allows for the identification of the main components of the resin, such as naphthalene, phenanthrene, pyrene, and their derivatives; GLC provides high resolution for separation and identification of concentrations of volatile and semi-volatile compounds.

The main purpose of this work was to evaluate the applicability of Qarmet coal tar for the production of electrode pitch. GLC was chosen as the most accessible and widely used method for the analysis of volatile and semi-volatile resin components. The data obtained allow us to draw important conclusions about the potential of using Qarmet coal tar to produce electric pitch.

**Figure 1:**
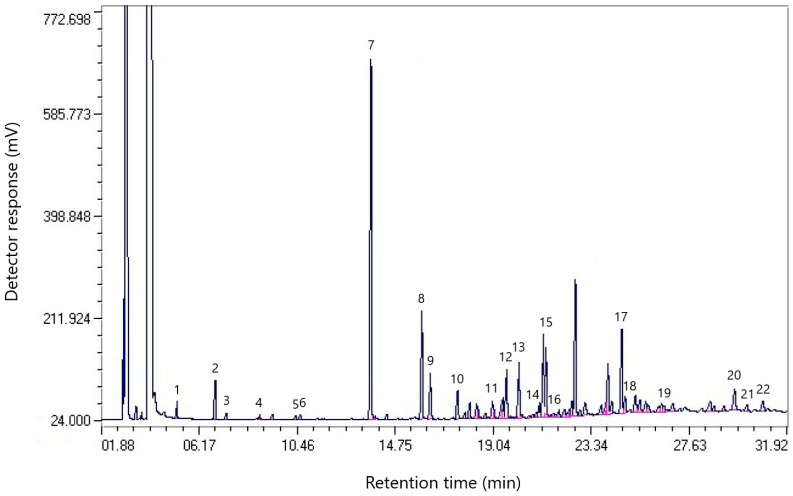
Chromatogram of the component composition of the resin sample from dump № 1.

The chromatogram of the component composition of coal tar from dump № 1 is shown in Figure 1.

Table 1 shows the component composition of the coal tar sample from dump № 1.

Table 1 shows that naphthalene accounts for the largest share of coal tar from dump № 1 (19.86%), which indicates a high concentration of polycyclic aromatic hydrocarbons (PAHs). A significant content of its derivatives includes 1-methylnaphthalene (6.10%) and 2-methylnaphthalene (2.75%), which indicates the potential suitability of the resin for processing into carbon materials. Other significant components include phenanthrene (5.54%), fluorene (4.34%), dibenzofuran (3.61%), and acenaphthene (3.40%), which can be used to produce pitch and other carbon products.

The composition contains oxygen-containing compounds, such as myristic acid (0.91%), palmitic acid (0.74%), and stearic acid (0.63%), which indicates the possible presence of resin oxidation processes under storage conditions [19].

The total number of identified compounds was 57.84%, which demonstrates the complex multicomponent nature of the resin.

The presence of high-boiling compounds, such as fluoranthene (1.95%) and pyrene (1.05%), indicates the possibility of obtaining materials with high temperature resistance.

**Figure 2:**
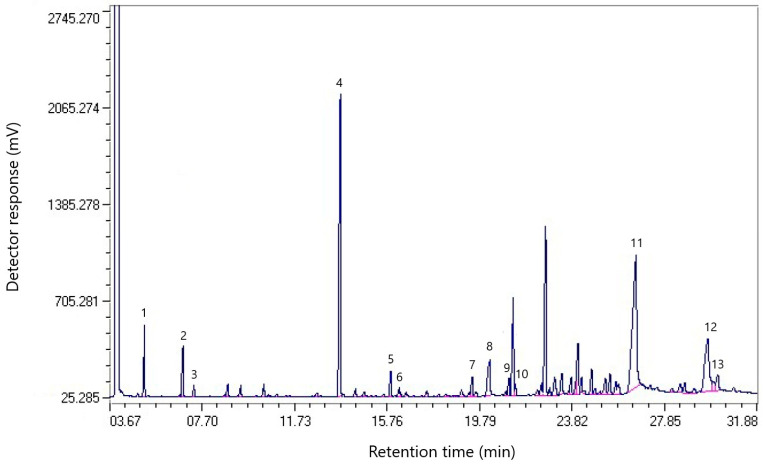
Chromatogram of the component composition of the resin sample from dump № 2.

Figure 2 shows the chromatogram of the component composition of a sample of coal tar from dump № 2.

Table 2 shows the component composition of coal tar from dump № 2.

According to the data in Table 2, it can be noted that, unlike the sample from dump № 1, which contains more polycyclic aromatic hydrocarbons (naphthalene—19.86%, phenanthrene—5.54%), the sample from dump № 2 contains more fatty acids (palmitic acid—22.43%; oleic acid—9.95%; lauric acid—3.57%; stearic acid—1.54%), which may indicate an intensive course of oxidative processes in the resin. These acids are saturated and unsaturated carboxylic compounds, and their presence indicates possible oxidation of the resin during storage.

Table 2 shows that the main aromatic component of coal tar from dump № 2 is naphthalene (15.47%), which indicates its high potential for processing into valuable products. The relatively low boiling temperature (~218 °C) makes it possible to efficiently extract it from the resin by distillation [20].

Coal pitch is a by-product of coal coking; therefore, it has a good affinity for coal and can effectively improve the quality of coke as a binder. However, it ages easily during storage, thereby losing lean substances and forming asphaltenes [21,22]. Asphaltenes can be formed as a result of polycondensation and aromatization of hydrocarbons in coal tar after removal of volatile substances. During the long period that the resin was in the settling tanks, changes in the physical properties occurred, and the GLC analysis showed some impurities, presumably various oils from the sheet rolling mill, and, consequently, resin samples from landfills № 1 and № 2 are not suitable for obtaining pitch of a given class “V”.

**Figure 3:**
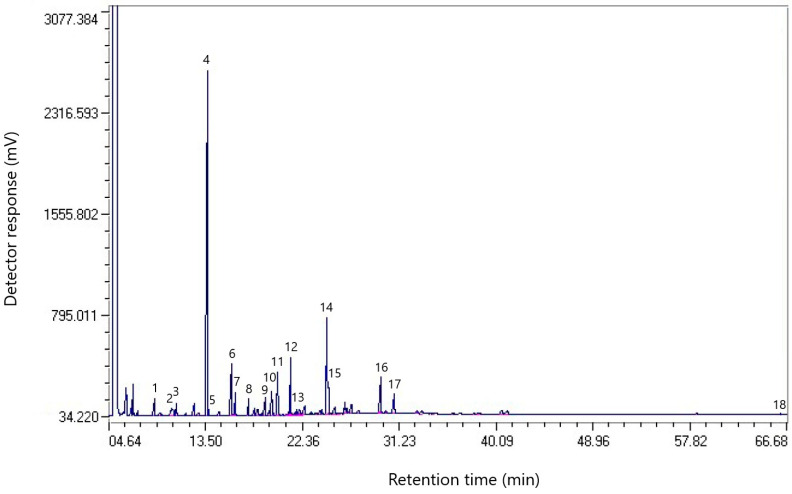
Chromatogram of the component composition of a coal tar sample taken from a coke plant.

Figure 3 shows the chromatogram of the component composition of a sample of coal tar taken from a coke chemical plant.

Table 3 shows the component composition of the resin sample taken from the coke chemical plant.

Analysis of the data presented in Table 3 shows that the coal tar sample taken from the coke plant contains a significant proportion of polycyclic aromatic compounds: naphthalene (31.48%), phenanthrene (8.63%), fluorene (4.63%), fluoranthene (4.56%), pyrene (2.84%), and anthracene (2.29%). There are also heterocyclic compounds: dibenzofuran (3.60%) and 2-Benzothiophene (0.48%).

To obtain pitch, coal tar obtained directly from the production of coke (not from landfills) from the hard coals of the Karaganda coal basin was used. As a result of distillation, the yield of coal pitch during vacuum distillation of Qarmet coal tar weighing 200 g was 90 g (45%).

Table 4 shows the qualitative characteristics of the Qarmet coal pitch obtained by vacuum distillation.

The composition of the fraction in baking soda insoluble in toluene and quinoline was determined using the GLC analysis method.

**Figure 4:**
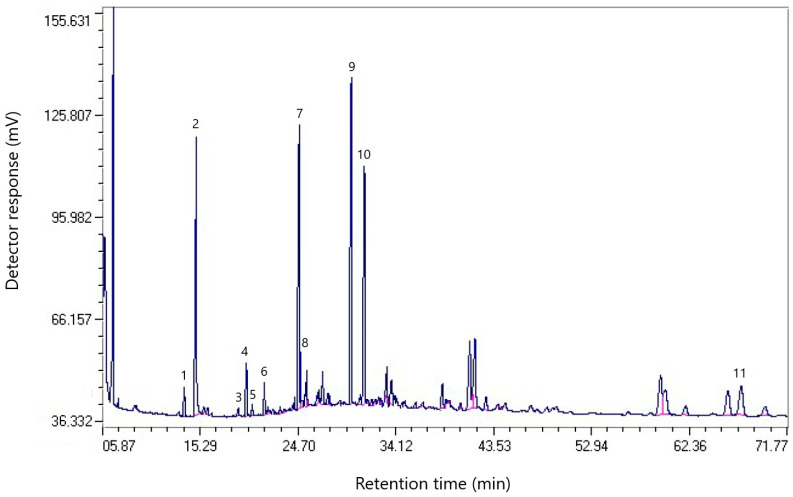
Chromatogram of the composition of fractions of Qarmet pitch soluble in toluene.

The chromatogram of the composition of pitch fractions obtained by distillation of coal tar from the Qarmet coke chemical production, soluble in toluene, is shown in Figure 4.

The composition of fractions of pitch obtained by distillation of coal tar from the coke chemical production of Qarmet, soluble in toluene, is shown in Table 5.

**Figure 5:**
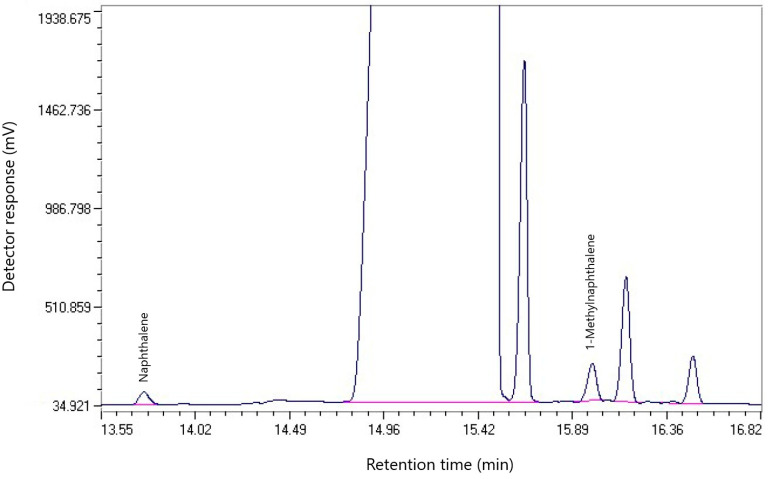
Chromatogram of the composition of Qarmet pitch fractions soluble in quinoline.

Figure 5 shows a chromatogram of the composition of Qarmet pitch fractions soluble in quinoline.

The composition of pitch fractions obtained by distillation of coal tar “Qarmet”, soluble in quinoline, is shown in Table 6.

One of the most important technological parameters characterizing the properties of pitch is the softening temperature *T_s_*. The softening temperature is an important parameter that determines the conditions of processing and use of pitch, affecting processes such as pressing and heat treatment, which determine the final properties of carbon materials [23]. Depending on the temperature, baking is divided into mild (40–55 °C), medium-temperature (65–90 °C), and high-temperature (135–150 °C). The resulting pitch during the distillation of Qarmet coal tar is classified as medium-temperature. Coal pitch, which has a softening temperature of 65–90 °C, is most widespread in the electrode and electric coal industries.

Also, the important characteristics of pitch used to produce electrical products (pitch grade “V” according to GOST 10200-2017) include the toluene-insoluble content, which should correspond to at least 31% [24]. According to the data presented in Table 4, the content of toluene-insoluble substances in the resulting baking was 41.86%, which is higher than the minimum level of at least 31% established by the standard.

Table 7 shows the material balance of the vacuum distillation process of coal tar obtained directly from coke production (not from landfills).

Thus, the quality of coal pitch obtained by vacuum distillation of Qarmet coal tar, taken directly from coke production (not from landfills № 1 and № 2), meets the requirements in terms of such basic indicators as the mass fraction of substances insoluble in toluene, softening temperature, volatile substances release, and ash content, requirements for furnaces for electrode production in accordance with GOST 10200-2017. The sample of the obtained Qarmet pitch corresponds to the “V” brand of the electrode pitch to a greater extent and is suitable for the production of electrode materials.

The content of quinoline-insoluble substances in coal tar is 21.05%, which indicates the need for further research and optimization of the conditions for obtaining and processing pitch to improve its characteristics.

Reducing the content of substances insoluble in quinoline improves the quality of pitch. The solution to this problem is possible by applying additional pitch treatment methods with various solvents. For example, in the work [25], it was found that the treatment of pitch with a mixture of coke-washing oil and aviation kerosene (in various ratios), using the method of distillation with solvent deposition, reduces the content of substances insoluble in quinoline in coal tar to 0.8%.

GLC analyses have shown that the total number of unidentified components in resin samples and vacuum distillation products is quite large, and this may be due to the complex composition of the raw materials, since coal tar is a multicomponent system containing thousands of different compounds, including aromatic, heterocyclic, oxygen-containing, and high-molecular-weight substances. Many of these compounds are poorly detected or not separated by gas–liquid chromatography (GLC). The GLC method is effective for the analysis of volatile and semi-volatile compounds of low molecular weight but less suitable for the analysis of high molecular weight and thermolabile substances. This leads to the fact that a significant part of the composition remains unidentified. The resin samples from landfills № 1 and № 2 contain impurities (for example, oils from rolling mills), as well as oxidation products (for example, fatty acids). These compounds may be mistakenly counted as “unidentified components”.

Despite the limitations of the GLC method, it has successfully identified the main components of the resin (e.g., naphthalene, phenanthrene, pyrene) and estimated their concentrations. These data are sufficient to characterize the quality of the obtained pitch and its compliance with standards.

## 3. Materials and Methods

### 3.1. Methods of Analysis

To determine the component composition of the organic component of Qarmet coal tar, the component composition was analyzed using gas–liquid chromatography (GLC). The GLC methods for analyzing the component composition of the organic mass of coal tar samples taken directly from the production of coke and Qarmet chemical dumps were used by us for the first time, since no one had previously conducted such studies.

The analysis of the organic products of the presented samples was carried out via GLC analysis on a Crystallux 4000 M chromatograph (NPF Meta-chrome, Yoshkar-Ola city, Russia) with a PID/PID detector module on a ZB-5 column of 30 m × 0.32 mm × 0.25 microns. The chromatograms were processed using the NetChrom v2.1 program.

### 3.2. Pitch Obtaining

Qarmet coal tar weighing 200 g was placed in a 500 mL double-necked flask equipped with a Wurtz nozzle, a capillary, an air cooler, an along for vacuum distillation and a receiving flask. The bulb was heated using a 1000 Watt bulb heater. The reduced pressure was created by a circulating vacuum pump SHZ-D-(III). The products were distilled with a gradual increase in the temperature of the flask heater in the range of 100–450 °C and a pressure of 8–10 mmHg. As it heats up, slightly boiling fractions are removed from the resin, and pitch accumulates in the flask. To extract the pitch, the flask is heated to a temperature of 250 °C, and the pitch is poured into a receiving container.

### 3.3. Determination of the Main Characteristics of Pitch, Determination of the Composition of the Fractions Dissolved in Toluene and Quinoline

The main characteristics that determine the quality of coal pitch for electrode production include softening temperature, solubility in toluene and quinoline, ash content, and the yield of volatile substances, which are included in GOST 10200-2017. These technological parameters make it possible to evaluate the suitability of the pitch for use in a particular field of application. The softening temperature of the pitches was determined according to GOST 9950-2020 [26]. The softening temperature (*T_s_*) was determined using the “Ring and rod” method [26].

The content of substances insoluble in toluene (α fractions) in coal tar and pitch was determined in accordance with GOST 7847-2020 [27]. According to GOST 10200-2017, the mass fraction of substances insoluble in quinoline (α1 fractions) was determined. The yield of volatile substances was determined according to GOST 70547-2022 [28]. The determination of the ash content of coal tar and pitch was carried out in accordance with GOST 70542-2022 [29]. The composition of fractions in baking soda soluble in toluene and quinoline was determined using the GLC analysis method.

## 4. Conclusions

In the course of the research, coal tar was distilled from the Qarmet coke chemical production in order to obtain medium-temperature coal pitch. The resulting pitch is characterized by the following basic physico-chemical parameters:

Softening temperature: 85–91 °C, which meets the requirements for electric pitch grade “V” according to GOST 10200-2017.

The content of substances insoluble in toluene is 41.86%, which is higher than the minimum level established by the standard (at least 31%).

Volatile matter yield: 55%, which meets regulatory requirements.

Ash content: 0.04%, which is significantly lower than the maximum allowable value of 0.3%.

The analysis of the component composition of coal tar using gas–liquid chromatography (GLC) showed that the main compounds are polycyclic aromatic hydrocarbons, such as naphthalene, phenanthrene, fluorophthene, and pyrene. These compounds give the pitch the necessary physico-chemical properties to ensure its suitability for use in the electric coal industry.

The fractional composition of pitch soluble in toluene indicates the presence of high-molecular-weight hydrocarbons, such as fluoranthene (11.71%), pyrene (10.13%), and benzopyrene (4.84%). This indicates the stability of the material and its potential for use as a binding component in the production of carbon materials.

Thus, this study showed that the coal pitch obtained from Qarmet coal tar meets the requirements of the “V” grade electrode pitch and can be used in the production of anodes for the aluminum industry. However, in order to improve the characteristics and ensure the stability of properties, further studies are planned to optimize the conditions for processing Qarmet coal tar. In the future, we plan to study the possibility of using the obtained pitch to produce electrode coke, which is a key material for the production of anodes in the aluminum industry. The influence of the composition and properties of pitch on the quality of the coke produced, including its density, mechanical strength, and electrophysical properties, will be investigated.

## Figures and Tables

**Table 1:** Component composition of the resin sample of dump № 1.

№	Compound	Retention Time, Min	Concentration, %
1	Toluene	5.21	0.57
2	m-Xylene	6.9	1.75
3	o-Xylene	7.35	0.27
4	Phenol	8.87	0.22
5	Indane	10.39	0.17
6	Indene	10.6	0.18
7	Naphthalene	13.69	19.86
8	1-Methylnaphthalene	15.94	6.1
9	2-Methylnaphthalene	16.33	2.75
10	Biphenyl	17.48	1.73
11	Acenaphthylene	19.06	0.61
12	Acenaphthene	19.61	3.4
13	Dibenzofuran	20.15	3.61
14	Myristic acid	21.05	0.91
15	Fluorene	21.33	4.34
16	Dihydroanthracene	21.77	0.16
17	Phenanthrene	24.64	5.54
18	Anthracene	24.78	1.2
19	Palmitic acid	26.49	0.74
20	Fluoranthene	29.59	1.95
21	Stearic acid	30.1	0.63
22	Pyrene	30.79	1.05
23	Identified		57.74

**Table 2:** Component composition of the resin sample from dump № 2.

№	Component	Retention Time (min)	Concentration (%)
1	Toluene	5.21	1.93
2	m-Xylene	6.89	1.85
3	o-Xylene	7.37	0.42
4	Naphthalene	13.7	15.47
5	1-Methylnaphthalene	15.93	1.13
6	2-Methylnaphthalene	16.32	0.2
7	Acenaphthene	19.45	1.44
8	Lauric Acid	20.15	3.57
9	Myristic Acid	21.05	0.84
10	Fluorene	21.37	0.63
11	Palmitic Acid	26.64	22.43
12	Oleic Acid	29.84	9.95
13	Stearic Acid	30.12	1.54
14	Identified		61.4

**Table 3:** Component composition of the resin sample from the coke chemical enterprise.

№	Compound	Retention Time (min)	Concentration (%)
1	Phenol	8.83	1.48
2	Indane	10.44	0.58
3	Indene	10.56	0.52
4	Naphthalene	13.68	31.48
5	2-Benzothiophene	13.82	0.48
6	1-Methylnaphthalene	15.86	4.19
7	2-Methylnaphthalene	16.23	1.9
8	Biphenyl	17.42	1.39
9	Acenaphthylene	18.94	1.53
10	Acenaphthene	19.56	2.1
11	Dibenzofuran	20.1	3.6
12	Fluorene	21.28	4.63
13	Dihydroanthracene	21.72	0.12
14	Phenanthrene	24.6	8.63
15	Anthracene	24.74	2.29
16	Fluoranthene	29.5	4.56
17	Pyrene	30.72	2.84
18	Benzopyrene	66.02	0.56
19	Identified		72.88

**Table 4:** Physico-chemical parameters of the Qarmet coal pitch.

Indicator Name	Standard for Grade “V”	Pitch Made from Coal Tar Obtained Directly from Coke Production (Not from Landfills)
Mass fraction of substances insoluble in quinoline, %, not more than	12	21.05
Mass fraction of substances insoluble in toluene, %, not less than	31	41.86
Softening temperature, °C, not more than	85–90	88
Volatile matter yield, %	53–57	55
Ash content, %, not more than	0.3	0.04
Mass fraction of water in solid pitch, %, not more than	4.0	~1.5

**Table 5:** Composition of fractions of Qarmet pitch soluble in toluene.

№	Component	Retention Time (min)	Concentration (%)
1	Naphthalene	13.79	0.67
2	Biphenyl	17.41	0.015
3	Acenaphthylene	18.96	0.23
4	Acenaphthene	19.75	1.32
5	Dibenzofuran	20.31	0.29
6	Fluorene	21.48	0.88
7	Phenanthrene	24.79	7.31
8	Anthracene	24.94	1.58
9	Fluoranthene	29.8	11.71
10	Pyrene	31.06	10.13
11	Benzopyrene	67.23	4.84
12	Identified		38.98

**Table 6:** Composition of fractions of pitch “Qarmet” soluble in quinoline.

Component	Retention Time (min)	Concentration (%)
Naphthalene	13.83	2.97
1-Methylnaphthalene	16.0	7.65
Identified		10.62

**Table 7:** Material balance of the vacuum distillation process of coal tar obtained directly during coke production (not from landfills).

Component	Mass, g	Mass Fraction, %
Coal tar	200	100
Light Fractions (Volatile)	110	55
Pitch	90	45

## Data Availability

Data are contained within the article.
